# Case Report: Fatal *Streptococcus pyogenes* infection secondary to closed femoral fracture

**DOI:** 10.3389/fmed.2026.1795714

**Published:** 2026-03-04

**Authors:** Danni Zhong, Jiajie He, Jie Jin, Yunping Pan

**Affiliations:** 1 Clinical Pharmacy Department, Ningbo No. 6 Hospital, Ningbo, China; 2 Department of Intensive Care Unit, Ningbo No. 6 Hospital, Ningbo, China

**Keywords:** closed fracture, multiple organ dysfunction syndrome, necrotizing fasciitis, streptococcal toxic shock syndrome, *Streptococcus pyogenes*

## Abstract

**Background:**

Invasive *Streptococcus pyogenes* infection is exceedingly rare in patients with closed fractures. However, it can precipitate life-threatening necrotizing soft-tissue infection and streptococcal toxic shock syndrome once it occurs. Early recognition and aggressive multidisciplinary management are essential for survival.

**Case summary:**

We presented a patient who sustained closed fracture of the upper segment of the left femur due to a traffic accident. He had a history of acute pharyngitis 10 days ago. The patient experienced fever on the second day after admission, with his body temperature ranging from 38.5 °C to 38.8 °C. He developed rapidly progressive swelling and severe pain at the fracture site on the third day, accompanied by high fever of 40.1 °C, tachypnea, and tachycardia, with mottled rash over the local skin, followed by hypotension, oliguria and a rise of serum creatinine. Emergent fasciotomy and decompression were performed, and the thigh musculature of patient was dusky-red and non-viable. Debridement was performed to remove the necrotic tissue. Subsequently, the patient rapidly developed to multiple organ dysfunction with profoundly elevated inflammatory markers. Thigh amputation surgery was undertaken to save his life. The wound secretion, blood culture, and plasma next-generation sequencing (NGS) all confirmed a diagnosis of streptococcal infection. The patient then underwent several times of debridement to remove necrotic tissue. Meanwhile, he took sensitive antibiotic therapy, aggressive fluid resuscitation and organ function support. Ultimately, his condition improved.

**Conclusion:**

This case underscored the catastrophic potential of closed fractures complicated by necrotizing soft tissue infections caused by *S. pyogenes*, highlighting the critical need for heightened vigilance in patients with a recent history of *Streptococcus* exposure. Early and aggressive surgical intervention, along with multidisciplinary collaboration, is essential. Additionally, the role of prompt pathogen culture testing and advanced diagnostic techniques, such as NGS, in pathogen identification is emphasized.

## Introduction

*Streptococcus pyogenes*, also known as group A *β*-hemolytic streptococcus, primarily colonizes the throat and is the most pathogenic member of the group. The infection can be classified into invasive infection and non-invasive infection. Non-invasive infections caused by *S. pyogenes* are typically mild and commonly present as upper respiratory tract infections and skin and soft-tissue infections. In contrast, patients with invasive infections have a dangerous condition, which may lead to necrotizing fasciitis (NF) and streptococcal toxic shock syndrome (STSS). The symptoms include rapid and progressive necrosis of the skin and soft tissues, accompanied by severe pain and systemic toxic symptoms. And the characteristics include rapid onset of hypotension and multiple organ failure. These patients often have no clear infection site, or clinical manifestations that are disproportionate to the original mild infection site. It is a severe and rapidly progressing disease, with an extremely high mortality rate (1, 2). Epidemiological studies indicated that the annual incidence of invasive group A *Streptococcus* (GAS) infection was approximately 3 cases per 100,000 population in developed countries, with 13%–15% of cases progressing to STSS. Despite aggressive treatment, the case fatality rate of STSS could still be as high as 23%–44% (3, 4). A study conducted abroad indicated that among the patients included in the research, the main pathogens causing infection related to fractures were *Staphylococcus aureus* (31.4%), *Staphylococcus epidermidis* (25.8%) and non-*epidermidis* coagulase-negative staphylococci (18.0%) (5). Other studies have shown that the proportion of *Streptococcus* species in fractures-related infections was approximately 5% (6). Cases of closed fracture complicated by invasive GAS infection leading to NF and STSS were extremely rare (7).

Pathophysiologically, bacterial invasion and the release of superantigens trigger an overwhelming, dysregulated immune-inflammatory response, leading to capillary leak, shock, and end-organ damage (8). Diagnosis relies chiefly on detailed medical history, clinical recognition of the rapid evolution of pain, swelling, mottled rash, and systemic toxic symptoms, supported by early microbiological confirmation. Usually, *Streptococcus* is isolated from sterile sites, accompanied by manifestations of two or more organs damage, such as hypotension, renal dysfunction, abnormal coagulation or thrombocytopenia, liver dysfunction, acute respiratory distress, soft tissue necrosis, etc. Traditional pathogen culture remains the gold standard for diagnosis, and molecular techniques like next-generation sequencing (NGS) can also provide rapid and comprehensive pathogen identification. Prompt administration of appropriate antibiotics and emergent, thorough surgical debridement of necrotic tissue are the cornerstones of management (9, 10). Delayed surgical intervention is a well-established predictor of poor outcomes.

Here, we reported a critical case of a young male patient who developed fatal necrotizing soft-tissue infection (NSTI) and STSS caused by GAS after a closed femoral fracture, ultimately requiring amputation and multiple debridement procedures to completely remove the necrotic tissue to save his life. The patient had a history of “acute pharyngitis” 10 days prior to this trauma. He did not continue the treatment after sore throat symptoms alleviated. The uniqueness of this case lies in the fact that the patient only had a closed femoral fracture without any open wounds, but eventually rapidly progressed to invasive GAS infection and STSS. It could not be excluded that this patient’s disease progression was related to the recent infection. Such an infection was extremely rare in closed fractures. This case suggested the possibility that patients with a recent history of infection might develop invasive GAS bloodstream infection after closed fractures. It emphasized the need of being vigilant for GAS NSTI and STSS when encountering non-traumatic common symptoms in the field of trauma orthopedics. Early identification and aggressive multidisciplinary management are important, and detailed history collection is also indispensable.

### Case report

**Figure 1:**
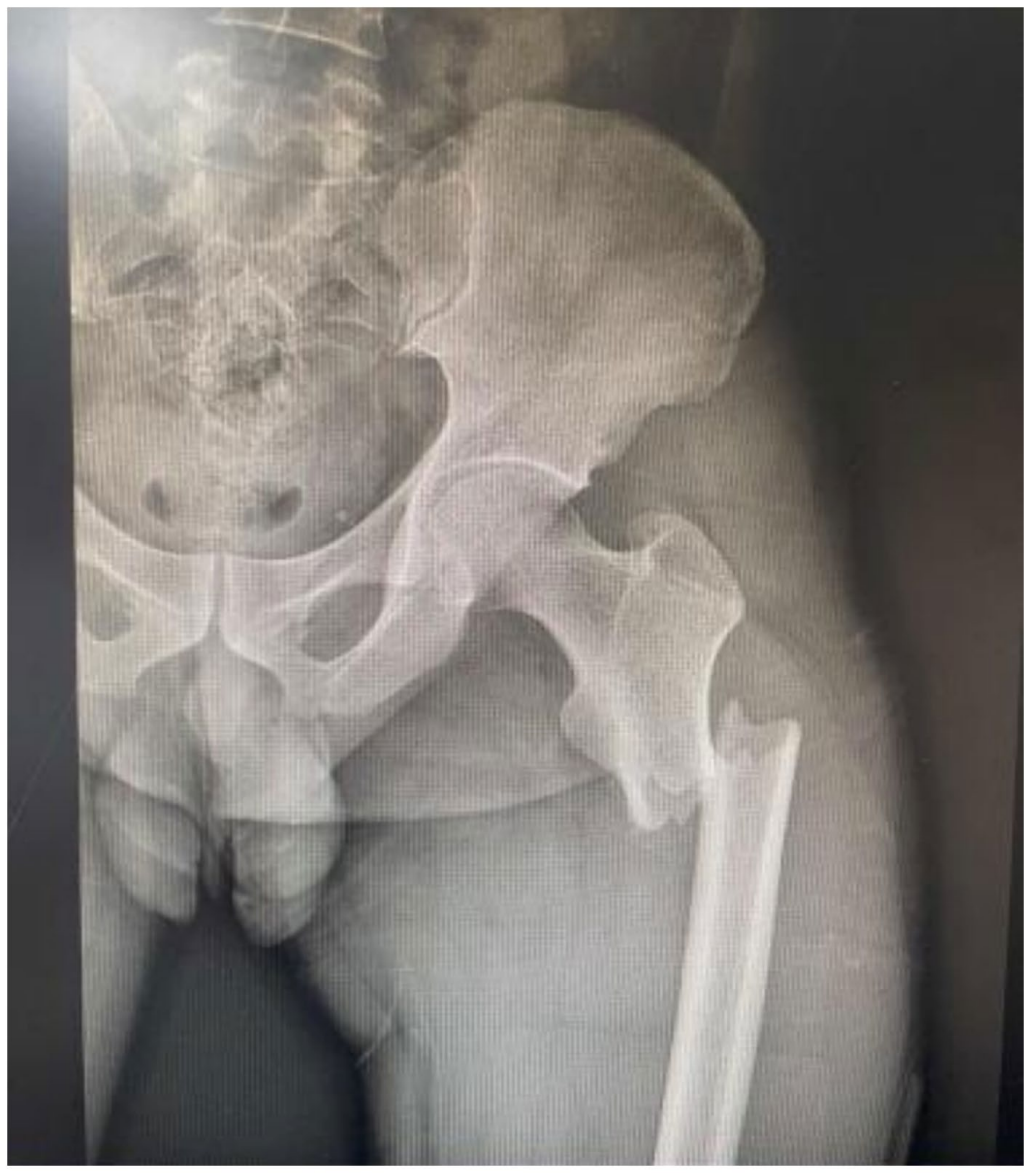
X-ray film of femoral fracture of the patient.

**Figure 2:**
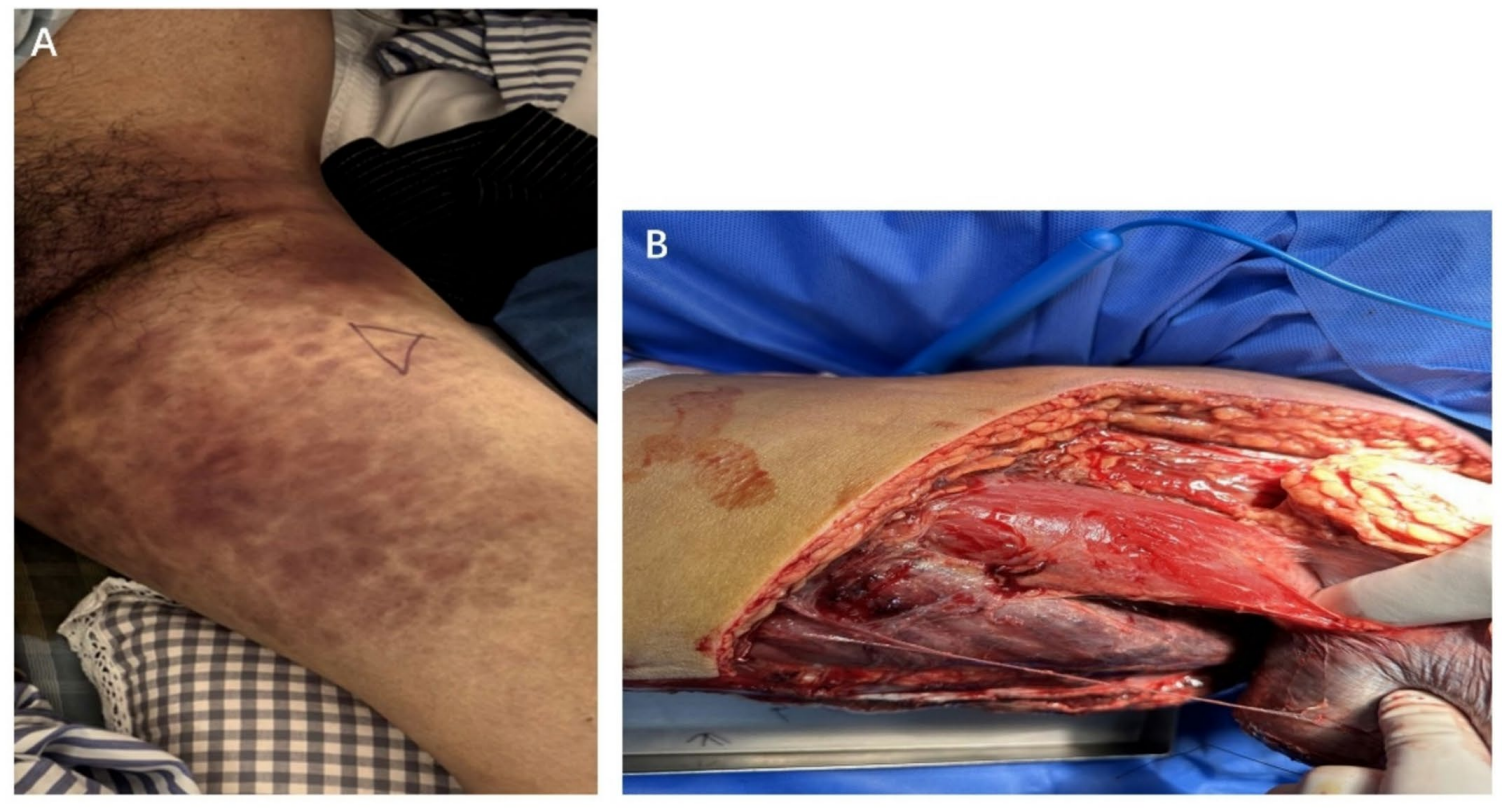
**(A)** The patient rapidly developed severe swelling and intense pain in the left thigh, and mottled, purplish skin rash appeared on this thigh. **(B)** The lateral thigh muscle was found to be dark red, non-viable, inelastic, and non-bleeding, with necrotic fascia intraoperatively.

**Figure 3:**
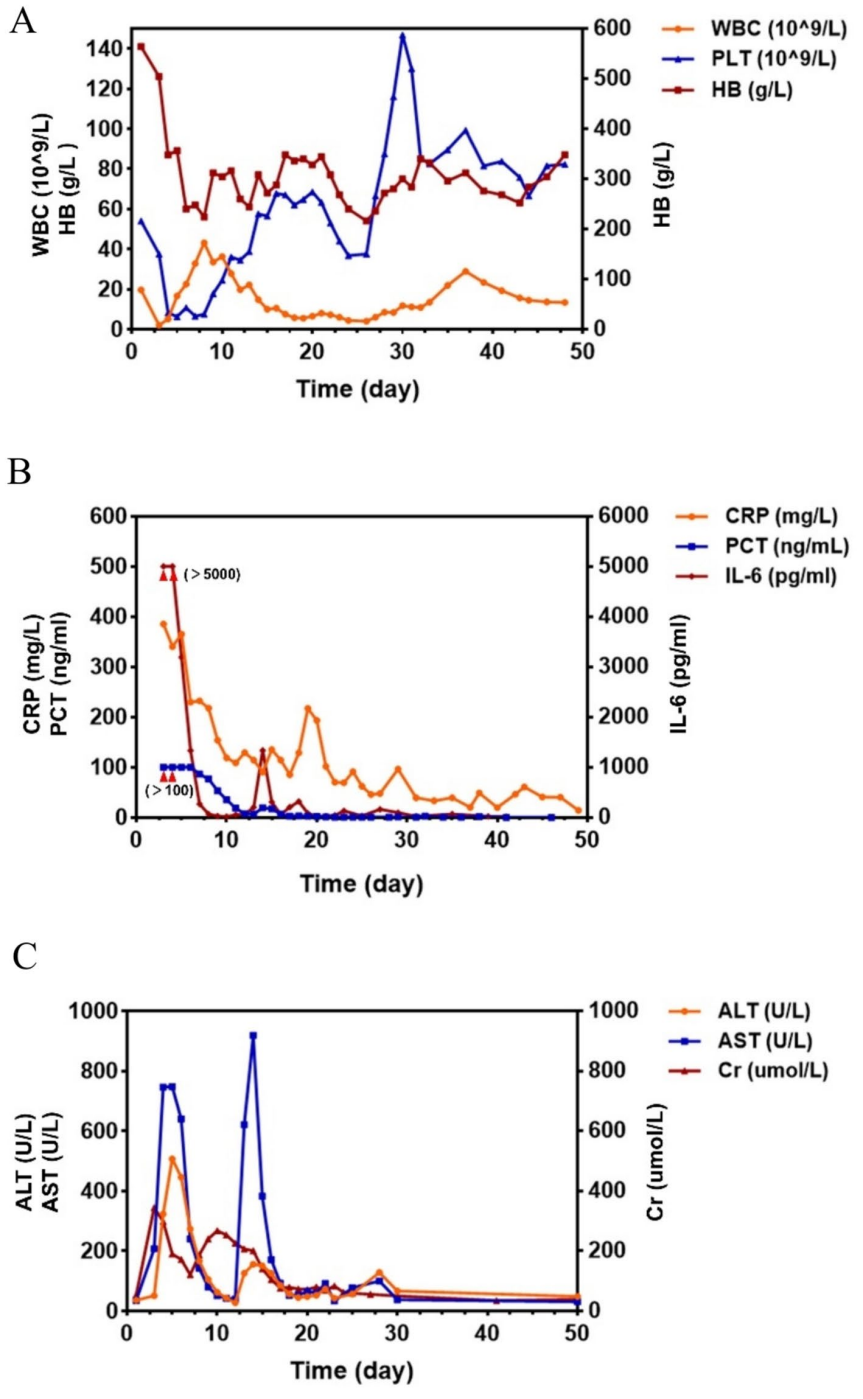
Curves of the blood count, inflammatory indicators, and liver and kidney function indicators during the patient’s treatment process. **(A)** Trends in blood counts throughout the course of treatment. **(B)** Trends in inflammatory indicators during treatment. **(C)** Trends in liver and kidney function indicators during treatment.

A 35-year-old male, a company employee, with a history of penicillin allergy was admitted to the trauma orthopedics department in our hospital on October 15, 2025 due to a closed left femoral upper segment fracture caused by a traffic accident (Figure 1). He had no smoking or drinking habits. He had no history of serious diseases such as malignant tumors, diabetes, or chronic skin diseases. And he denied a history of long-term medication use. The initial vital signs of this patient were stable upon admission. The patient experienced fever on October 16, with his body temperature ranging from 38.5 °C to 38.8 °C. His clinical condition deteriorated sharply on October 17. The patient presented with severe swelling and intense pain in the left thigh, accompanied by mottled, purplish skin rash (Figure 2A), high fever (40.1 °C), tachycardia (heart rate: 155 bpm), tachypnea (respiratory rate: 35 breaths/min), and hypotension (blood pressure: 80/55 mmHg). Subsequently, the patient developed oliguria and progressed to anuria. Laboratory findings revealed inflammatory indicators significantly increased, initial peripheral blood leukocytosis that rapidly progressed to leukopenia (white blood cell count (WBC): 19 × 10^9^/L to 2.1 × 10^9^/L), markedly elevated C-reactive protein (CRP: 382 mg/L), interleukin-6 (IL-6) levels >5,000 pg./L, procalcitonin >100 ng/L, acute renal failure (serum creatinine: 419.5 μmol/L), and metabolic acidosis (blood lactic acid: 12.07 mmol/L). At the same time, the patient presented with hyperkalemia (blood potassium level: 5.7 mmol/L), creatine kinase (CK) for 20,540 U/L and cardiac function impairment (troponin I: 4.17 ng/mL, brain natriuretic peptide 1810 pg./mL). The key indicators and the changes during the subsequent treatment process were shown in Figure 3.

The patient presented with symptoms of septic shock and multiple organ damage (circulation, respiration, kidneys, heart, internal environment). And compartment syndrome was strongly suspected; however, the infection site of septic shock is unclear. After blood culture specimen collection, immediate interventions were taken included the administration of broad-spectrum intravenous antibiotics (meropenem combined with linezolid, which was subsequently switched to daptomycin due to thrombocytopenia), aggressive fluid resuscitation for shock management, and correction of acidosis and hyperkalaemia. Emergent decompressive fasciotomy of the left thigh muscles and fascia was performed once vital signs had transiently stabilized. However, no obvious hematoma or active bleeding was observed during the operation, while the muscle compartments were found to be extremely tense, with portions of the fascia exhibiting necrosis. The vastus lateralis muscle was non-viable, appearing dark red, non-contractile, and non-bleeding, consistent with necrotizing fasciitis, myositis and myonecrosis (Figure 2B). Secretions for culture was also collected. During the operation, the family members were informed about the necrosis of the muscles and fascia observed. The original simple tension-reducing surgical method was changed, and the necrotic fascia and muscles were removed to expand the debridement. Despite these interventions, the patient’s systemic toxic response continued to worsen. Septic shock complicated by acute respiratory distress syndrome, acute renal failure with persistent anuria, circulatory failure, and sustained thrombocytopenia raised concerns for disseminated intravascular coagulation (DIC) and hepatic dysfunction. The patient required endotracheal intubation with mechanical ventilation to maintain oxygenation, high-dose norepinephrine (2.67 μg/kg min) to support circulation, and continuous renal replacement therapy. Clinically, ongoing tissue necrosis was suspected, necessitating further radical debridement. However, due to extremely unstable vital signs, the patient could not be transported for additional imaging evaluation of the affected limb to assess the extent of soft tissue necrosis. Ultrasonography of the left lower extremity revealed thrombosis in the proximal segment of the left superficial femoral vein. Given these factors, the trauma of repeated extensive debridement surgery would likely be intolerable for the patient, with substantial surgical risk. After repeated communication with the family, thigh amputation was performed in an effort to save the patient’s life and thoroughly eradicate necrotic tissue. Microbiological tests showed that Intra-operative wound discharge and two sets of paired blood cultures both grew *S. pyogenes* on October 19. The isolate was resistant only to erythromycin but remained susceptible to penicillins, quinolones, glycopeptides, and oxazolidinones. Subsequent plasma metagenomic next-generation sequencing also detected *S. pyogenes* on October 21. No virulence genes were detected, and no genetic typing was performed. The final diagnosis was STSS secondary to GAS NSTI.

**Figure 4:**
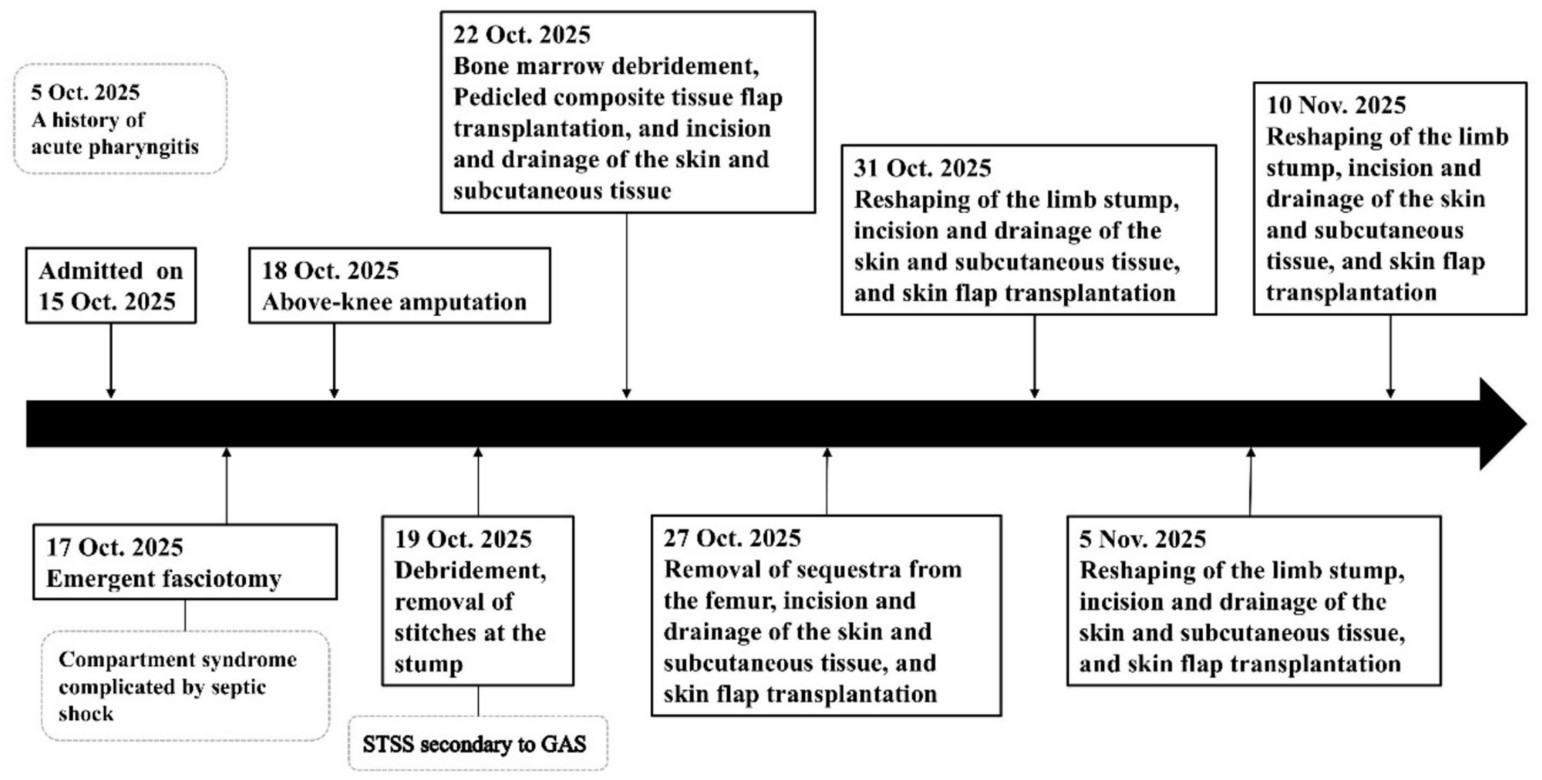
Main surgical treatment flowchart. The patient underwent amputation surgery, followed by multiple wound revisions, removal of residual necrotic soft tissues and bone tissues, and treatment with negative pressure closed drainage. STSS, streptococcal toxic-shock syndrome; GAS, group A *Streptococcus.*

Further history revealed that the patient had suffered from acute pharyngitis 10 days ago. The patient received cefmetazole 2.0 g intravenously once daily for 2 days at a local health clinic. After slight alleviation of sore throat symptoms, treatment was not continued. Subsequently, the patient’s sore throat symptoms resolved spontaneously. After 12 days of intensive care including pathogen-directed antibiotic therapy (due to secondary bacterial and fungal infections, antimicrobial therapy was adjusted based on susceptibility testing results to include meropenem, eravacycline, and fluconazole), nutritional support, repeated surgical debridement of residual necrotic soft tissue and bone, and vacuum-sealed drainage, the patient’s condition gradually stabilized, and his organ dysfunction resolved. Two weeks later, he was successfully weaned from mechanical ventilation and transitioned to high-flow nasal oxygen. Meanwhile, CRRT was discontinued after renal function recovered. Subsequently, he was transferred to the burn ward for definitive wound reconstruction (see Figure 4).

## Discussion

This case highlighted a rare and catastrophic complication of closed femoral fracture: invasive *S. pyogenes* infection resulting in necrotizing fasciitis and STSS with fulminant progression. We thought that the patient’s recent history of pharyngitis suggested a plausible pathogenic mechanism. Ten days prior to the trauma, the patient developed fever and sore throat and was diagnosed with upper respiratory tract infection and acute pharyngitis at a local health clinic. He received cefmetazole 2.0 g intravenously once daily for 2 days, and discontinued treatment after symptomatic relief. Clearly, there was inappropriate antimicrobial use and insufficient treatment duration. Although the patient’s symptoms subsequently resolved, this may have allowed the preceding *S. pyogenes* to persist as colonization. The recent episode of pharyngitis was not severe, and the patient had forgotten about this infection since the subjective symptoms had already resolved. Following the trauma, the resulting physiological stress and compromised immune function likely predisposed to bacteremia from the previously colonized bacteria, with subsequent hematogenous seeding at the fracture hematoma site (7). The hematoma served as an ideal culture medium for bacterial proliferation, triggering a destructive inflammatory cascade, and leading to the rapid outbreak of infection. In addition to presenting this case, we performed a comprehensive literature review on *S. pyogenes* infections.

*Streptococcus pyogenes*, is a common bacterium with significant invasive potential (1). After a period of relative stability, the global incidence of invasive GAS (iGAS) disease has recently resurged, showing marked fluctuations across multiple countries and regions (11). These variations are attributed to shifts in predominant M-protein *emm* types, changes in population immunity, and social or environmental factors, such as co-circulating viral respiratory infections. *Emm* typing remains the gold-standard method for characterizing GAS strains. Among these, *emm1*, *emm12*, *emm28*, and *emm89* represent the predominant global clones responsible for iGAS disease (12). Notably, the *emm1* genotype, particularly its hypervirulent sub-lineage M1UK, has been strongly associated with the increasing incidence of severe necrotizing fasciitis and STSS (12). Regrettably, the patient did not undergo *emm* typing. Early recognition remains one of the strongest modifiable predictors of survival. Rapid antigen detection tests (RADTs) for *S. pyogenes*, which offer high specificity and rapid results within minutes, have become the first-line screening tool for GAS pharyngitis in outpatient settings. However, due to their variable sensitivity, negative RADT results in high-risk patients still require confirmation by bacterial culture (13). Nucleic acid amplification methods, such as polymerase chain reaction (PCR), demonstrate superior sensitivity and specificity. These methods are increasingly being adopted for the rapid detection of GAS in throat swabs, sterile body fluids, and tissue samples, significantly reducing the diagnostic window (13). In difficult-to-diagnose or severe infections, metagenomic next-generation sequencing (NGS) provides an unbiased approach to pathogen identification, offering significant diagnostic value for sepsis of unknown origin or polymicrobial infections. In suspected necrotizing fasciitis, contrast-enhanced computed tomography (CT) or magnetic resonance imaging (MRI) may demonstrate characteristic findings such as fascial edema, fluid collections, and gas tracking. However, these imaging modalities lack 100% sensitivity. In this case, due to extremely unstable vital signs and acute renal failure, this patient did not undergo contrast-enhanced CT or MRI. Definitive diagnosis requires surgical exploration with tissue biopsy for Gram staining and culture, which remains the gold standard. This patient was ultimately diagnosed through surgical exploration, blood culture, and wound secretion culture.

*β*-lactam antibiotics, remains the cornerstone of therapy for GAS infections, with no clinically significant resistance reported to date (13). For iGAS infections, which are associated with high case-fatality rates, current guidelines recommend early combination therapy consisting of high-dose penicillin G plus clindamycin. Clindamycin provides critical adjunctive benefits by suppressing bacterial toxin production, modulating the host inflammatory response, demonstrating a prolonged post-antibiotic effect, and maintaining efficacy against stationary-phase bacteria (14). In penicillin-allergic patients, viable alternatives include vancomycin, linezolid, or daptomycin. This patient reported a history of penicillin allergy, developed acute renal failure, and subsequently presented with multi-organ failure and thrombocytopenia. Antimicrobial therapy was initiated with linezolid and later switched to daptomycin.

For NF, immediate and aggressive surgical debridement is life-saving, as delays can lead to catastrophic outcomes (14). For patients with closed fractures, differential diagnosis with osteofascial compartment syndrome (OCS) is essential. Both NF and OCS are catastrophic limb emergencies with highly overlapping early clinical manifestations, including severe pain, swelling, and functional impairment. However, their etiologies, pathophysiology, and treatment strategies differ fundamentally. OCS is a sterile ischemic condition caused by acute pressure elevation within closed osteofascial compartments, with muscle and nerve ischemia at its core, requiring emergent fasciotomy as the primary treatment (15, 16). In contrast, NF is a rapidly progressive invasive soft tissue infection characterized by fascial and muscular necrosis, systemic toxic shock, and high mortality, necessitating prompt radical debridement combined with potent antimicrobial therapy (17). In closed fractures, these two conditions are easily confused, making early accurate differentiation critical for limb salvage and survival (18, 19). In this case, the trauma orthopedist initially considered OCS and administered anti-edema treatment with observation for 1 day. However, by the third day, the patient developed shock, multiple organ dysfunction syndrome (MODS), and markedly elevated inflammatory markers. After multidisciplinary discussion, septic shock was suspected, and aggressive antimicrobial therapy with emergency surgery was undertaken. Intraoperative findings confirmed NF with necrosis of the lateral thigh muscle group. Song et al. reported a patient who was admitted with fever, swelling and pain of the left knee. STSS was diagnosed when the lesion rapidly extended and deteriorated, and he finally died of MODS. The case emphasized that even apparently localized GAS infection must be taken seriously. Patients whose condition deteriorated swiftly required early recognition, prompt intervention, and timely surgical debridement (20). The success of our case lay in the timely and decisive amputation performed after the initial debridement, when the patient’s condition rapidly deteriorated, following swift communication with the family, which eradicated the infectious focus and saved the patient’s life. Regrettably, when symptoms first appeared, the patient was observed for a day, after which the condition rapidly worsened, and the limb could not be salvaged. Niu et al. (21) reported a middle-aged man who underwent laparoscopic left adrenalectomy for a cortical adenoma. Within 24 h he developed pain and swelling around the surgical drain site, and the process progressed rapidly to necrotizing infection of the skin and subcutaneous tissues, septic shock and MODS. *Streptococcus pyogenes* was cultured from drain fluid and necrotic tissue. While comprehensive critical care, including antimicrobial therapy, CRRT and fluid resuscitation was provided, simultaneous aggressive surgical debridement and open drainage were performed. The patient finally recovered. Early and thorough debridement with adequate drainage was the critical determinant of therapeutic success. And Zhang et al. (22) showed a necrotizing fasciitis and myositis complicated by STSS case which caused by GAS. The patient underwent broad-spectrum antimicrobial therapy, debridement, supportive measures and amputation, and he eventually recovered and was discharged from the hospital, underscoring the importance of early recognition and invasive GAS infections management.

In STSS, intravenous immunoglobulin (IVIG) may mitigate the severe inflammatory cascade by neutralizing streptococcal superantigens (23). Although the evidence base is still evolving, current guidelines increasingly recommend IVIG for severe cases of STSS. No IVIG treatment was given for this case. iGAS infections, particularly STSS and necrotizing fasciitis, are associated with a poor prognosis. Even with optimal treatment, STSS mortality remains alarmingly high (30%–60%), with most fatalities occurring within 24–72 h of symptom onset, primarily due to refractory shock and multi-organ failure (14). Necrotizing fasciitis carries a mortality rate of 20%–40%, and survivors often experience significant long-term disability secondary to extensive tissue destruction. Several factors predict worse outcomes, including advanced age, multiple comorbidities, delayed diagnosis, failure to perform early surgical debridement, renal failure, and hypoalbuminemia (14). A recent nationwide multicenter retrospective cohort study from Japan demonstrated three independent high-risk predictive factors for the development of STSS in patients with GAS infection: (1) WBC count <4.0 × 10^9^/L; (2) CK ≥ 300 U/L; and (3) *emm1* genotype. These three markers showed a positive rate of 78.3% in fatal STSS cases. Patients with concurrent leukopenia, elevated CK, and *emm1* genotype had a 12.7-fold increased risk of developing STSS compared to those without these three indicators. Our patient, except for the absence of genetic typing, met the criteria for both leukopenia (2.1 × 10^9^/L) and markedly elevated CK (20,540 U/L), which is consistent with the findings of this study (24).

Our case underscored these key lessons. First, pain disproportionate to the apparent trauma, rapidly progressive soft-tissue swelling, cutaneous changes such as erythema and necrosis, and systemic signs of sepsis (fever, tachycardia, hypotension) should prompt immediate intervention (9). Second, if patients with GAS infection meet the following criteria: WBC count <4.0 × 10^9^/L, CK ≥ 300 U/L, *emm1* genotype, are highly indicative signs of STSS. Third, primacy of surgical intervention. While medical management (antibiotics, resuscitation, organ support) is essential, it alone cannot halt disease progression. Definitive treatment for NSTI requires prompt and radical debridement of all necrotic tissue (2, 9). In this case, despite fasciotomy within 24 h of symptom onset, the extensive necrosis necessitated amputation, underscoring the fulminant nature of GAS-associated NSTI. Additionally, role of advanced diagnostics. Although conventional cultures remain the diagnostic gold standard, molecular techniques like NGS offer rapid and comprehensive pathogen identification. This is particularly critical when cultures yield negative results but clinical suspicion remains high, enabling timely de-escalation or targeted therapy (25). Then, multidisciplinary collaboration. This near-fatal case was successfully managed through seamless coordination among orthopedic, intensive care, infectious disease and burn teams, as well as the clinical pharmacy department. Concurrent control of the infectious source and systematic organ support were pivotal to achieving a favorable outcome (26, 27). The comprehensive and detailed collection of a patient’s history clinically is of vital importance for the diagnosis of the disease.

This case also had its limitation. The disease progressed extremely rapidly; despite prompt treatment and interventions, the pace of deterioration exceeded all expectations. It showed the critical importance of heightened clinical vigilance. Orthopedic trauma teams must maintain a high index of suspicion for NSTIs, even following closed injuries. In our case, the patient did not undergo *emm* typing. For cases like this, it is necessary for us to conduct further pathogen analysis in the future studies.

## Conclusion

This report serves as a stark reminder that a closed fracture can become the nidus for life-threatening invasive streptococcal infection, especially when recent streptococcal disease has occurred. Early recognition of NSTI, immediate initiation of broad-spectrum antibiotics, and emergency radical surgical debridement are the key steps to improve survival in such patients. IGAS has re-emerged as a significant global public health threat. Clinicians must remain vigilant to its evolving epidemiology and the fulminant course of severe infections. While advances in diagnostic technologies like rapid molecular testing enable earlier intervention, outcomes ultimately depend on clinical suspicion and timely action. High-risk populations (e.g., diabetics, immunocompromised individuals, and patients with skin lesions) presenting with fever plus localized infection signs or systemic toxicity, with or without an obvious focus, require prompt consideration of iGAS. Management should follow the principle of early, high-dose, combination antibiotics, and timely surgery.

## Data Availability

The original contributions presented in the study are included in the article/supplementary material, further inquiries can be directed to the corresponding author.
